# Analysing the eosinophil cationic protein - a clue to the function of the eosinophil granulocyte

**DOI:** 10.1186/1465-9921-12-10

**Published:** 2011-01-14

**Authors:** Jonas Bystrom, Kawa Amin, David Bishop-Bailey

**Affiliations:** 1 Translational Medicine and Therapeutics, William Harvey Research Institute, Bart's and the London, Queen Mary University of London, Charterhouse Square, London EC1M 6BQ, UK; 2 Respiratory Medicine and Allergology, Department of Medical Science, Uppsala University Hospital, Uppsala, Sweden; 3 College of Medicine, Sulaimani University, Sulaimani, Iraq

## Abstract

Eosinophil granulocytes reside in respiratory mucosa including lungs, in the gastro-intestinal tract, and in lymphocyte associated organs, the thymus, lymph nodes and the spleen. In parasitic infections, atopic diseases such as atopic dermatitis and asthma, the numbers of the circulating eosinophils are frequently elevated. In conditions such as Hypereosinophilic Syndrome (HES) circulating eosinophil levels are even further raised. Although, eosinophils were identified more than hundred years ago, their roles in homeostasis and in disease still remain unclear. The most prominent feature of the eosinophils are their large secondary granules, each containing four basic proteins, the best known being the eosinophil cationic protein (ECP). This protein has been developed as a marker for eosinophilic disease and quantified in biological fluids including serum, bronchoalveolar lavage and nasal secretions. Elevated ECP levels are found in T helper lymphocyte type 2 (atopic) diseases such as allergic asthma and allergic rhinitis but also occasionally in other diseases such as bacterial sinusitis. ECP is a ribonuclease which has been attributed with cytotoxic, neurotoxic, fibrosis promoting and immune-regulatory functions. ECP regulates mucosal and immune cells and may directly act against helminth, bacterial and viral infections. The levels of ECP measured in disease in combination with the catalogue of known functions of the protein and its polymorphisms presented here will build a foundation for further speculations of the role of ECP, and ultimately the role of the eosinophil.

## Discovery of the eosinophils

Eosinophils were discovered in the blood of humans, frogs, dogs and rabbits in 1879 by Dr. Paul Ehrlich [1]. At that time, the German chemical industry was flourishing and Ehrlich took advantage of newly developed synthetic dyes to develop various histological staining techniques. The coal tar derived, acidic and bromide containing dye eosin identified blood cells containing bright red "alpha-granules" and the cells were named eosinophilic granulocytes. Due to the acidity of the staining solution Ehrlich could not at the time say with certainty that the eosinophilic granules contained protein, though he speculated that if present, protein might be denatured by the low pH of the dye [1]. Subsequently it was shown that eosin binds highly basic proteins which constitute the granules of these cells. These charged proteins are contained in on average twenty large granules dispersed throughout the cytoplasm of each cell, which the eosin stain awards the characteristic red spotted appearance that discriminates eosinophils from other leukocytes [2]. More than a century later the physiological roles of these granular proteins have yet to be fully identified.

In eosinophil granules pH is maintained at 5.1 by an ATPase [3] where the basic proteins are packed forming crystals [2]. The main content of these granules are four proteins, the major basic protein (MBP) present in their cores, surrounded by a matrix built up of eosinophil peroxidise (EPO), the eosinophil protein X/eosinophil derived neurotoxin (EPX/EDN) and ECP. Vesicotubular structures within the granules direct a differential release of these proteins [4]. The granule proteins were all discovered and characterised about one hundred years after the discovery of the eosinophils [5-8]. ECP is the best know of the proteins, assessed and used extensively as a marker in asthma and other inflammatory diseases. ECP has been scrutinized in a number of functional studies. The aim of this article is to review some of the findings of ECP quantifications in various diseases and set those in context of the experiments that have functionally analysed the protein. The findings will be used as guidance in a speculation of the biological role of eosinophil.

## ECP is mainly produced during the terminal expansion of the eosinophils in the bone marrow

**Figure 1:**
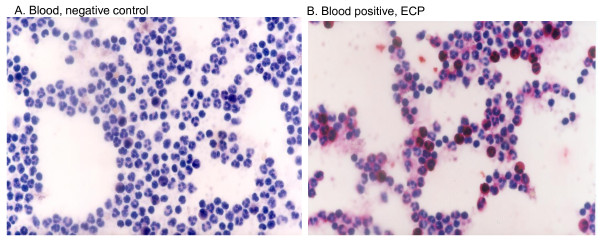
**Identification of eosinophil granulocytes in peripheral blood by immunohistochemical detection of ECP**. (A) Negative control (omission of primary antibody). Shown are peripheral leukocytes after fixation, incubation with alkaline phosphatase-anti-alkaline phosphatase (APAAP) with fast red substrate and counterstaining with Mayer's hematoxylin. The characteristic red immune-labelling reaction is absent. (B) Leukocytes are treated as in (A) but with addition of anti-ECP antibody. Peripheral leukocytes are visible but only the eosinophils have been stained for ECP. Original Magnification (X420).

Eosinophil progenitors (EoP's) in the bone marrow are the first cell identified exclusively of the eosinophil lineages. These EoP's express the cell surface markers IL-5R^+ ^CD34^+ ^CD38^+ ^IL-3R^+ ^CD45RA^-^, haematopoietic lineage associated transcription factor GATA-1, ECP mRNA transcripts and have visual characteristics of early eosinophilic blast cell [9,10]. Most of the granule protein production takes place as EoP's undergo the final stages of maturation [11,12]. ECP is synthesised, transported and stored in the mature secondary granules at such a high rate as that when the eosinophils are ready to leave the bone marrow, they contain 13.5 μg ECP/10^6 ^cells [13] (Figure 1B). Eosinophils are the major ECP producing cell while monocytes and myelo-monocytic cell lines produce minute amounts in comparison [14]. Activated [15] but not resting neutrophils also produce some ECP and have the ability to take up further ECP from the surrounding environment storing it in their azurophil granules [16,17]. In the myelo-eosinophilic cell line HL-60 clone 15, ECP production is dependent on a nuclear factor of activated T-cells (NFAT)-1 binding site in the intron of the ECP gene (denoted *RNASE3*) [18]. The *RNASE3 *gene was formed by gene duplication of an ancestral gene about 50 million years ago, the other duplication gene product being the eosinophil granule protein EPX/EDN gene (*RNASE2*). ECP and EPX/EDN are two ribonucleases with such a high degree of homology that they are unique to humans and primates and not found in other species. After this gene duplication however, ECP lost part of its ribonuclease activity, but acquired cytotoxic activity, whereas EDN/EPX remained a potent ribonuclease [19].

## ECP a cytotoxic ribonuclease

**Figure 2:**
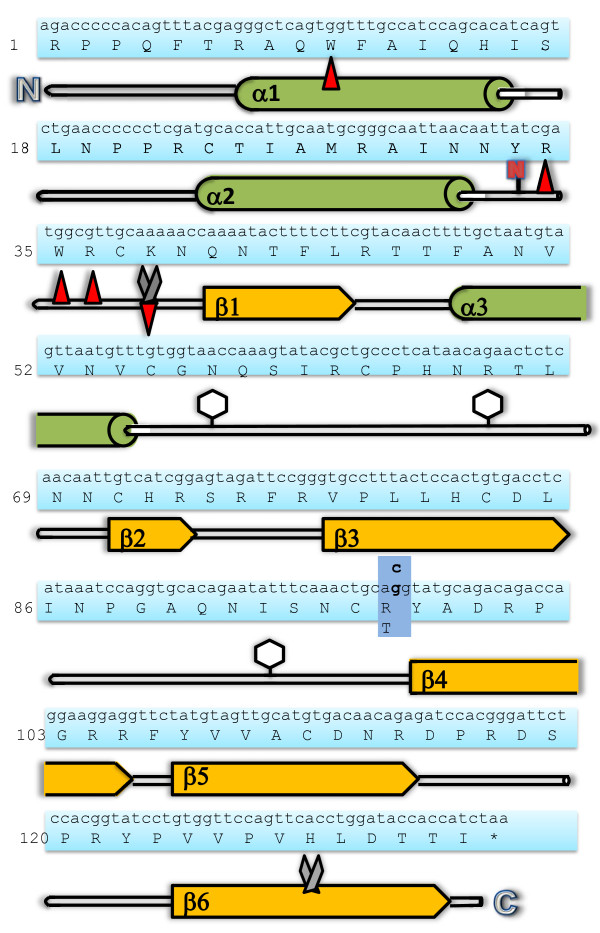
**The *RNASE3 *(ECP) gene and ECP protein sequence with numbers referring to the amino acid sequence**. Below the protein sequence is a schematic diagram of the peptide sequence where the beta sheet domains and the alpha helix domains are shown as red arrow and green barrel structures, respectively. Amino acids involved in RNase activity are represented by scissors. Amino acids involved in membrane interference, heparin binding and bactericidal activity are represented by red arrows. Glycosylated amino acids are represented with a glycomoiety while the letter N highlights the nitrated amino acid. A blue box shows the site of the amino acid altering polymorphism rs2073342.

ECP has homology to pancreatic ribonuclease and has the ability to degrade RNA [20]. The amino acid sequence of ECP has eight cysteine residues spaced all throughout the peptide establishing the tertiary structure of the protein by the formation of four cysteine double bonds. Two catalytic residues, a lysine and a histidine, responsible for the RNA degradation have been identified, K38 and H128 [20,21] (Figure 2) and these residues together with the cysteines are present in all members of the pancreatic ribonuclease family [20]. Analysis of the crystal structure of ECP verified this relationship to these other members of RNase family; namely a β-sheet backbone and three α-helices [22]. In a grove between two of the alpha helices the catalytic site for RNA degradation is located, with ECP showing a preference for cleaving poly-U RNA but not double-stranded RNA [23]. ECP consists of a single-chain peptide of 133 a.a. containing three sites for N-linked glycosylation, a.a.'s 57-59, 65-67 and 92-94 [24] (Figure 2). The glycosylation is composed of sialic acid, galactose and acetylglucosamine [25] explaining the variation in its detected size by Western blot of between 16 and 22 kDa [26]. Nineteen arginine residues facing the outside of the protein giving rise to the proteins basicity (pI > 11) [27] and possibly also its extraordinary stability compared to other ribonucleases [28]. In the presence of H_2_O_2 _ECP can be nitrated on tyrosine Y33 by EPO. This inflammation-independent nitration occurs during granule maturation and was suggested to enhance interactions after secretion between several of the otherwise repulsive, positively charged granule proteins (Figure 2) [29]. ECP has been shown to interact with artificial lipid membranes [30] and two tryptophan residues, W10 and W35 facing the outside, similar to the present arginine's, have been associated with this lipid membrane interaction [31]. ECP also has RNase independent cytostatic activity on tumour cells and the tryptophan residues contribute to this activity [32]. W35 was additionally found necessary for killing gram negative and gram positive bacteria [31]. The tryptophan's also facilitate ECP binding to heparin [33,34]. Another study found that the residues R34, W35, R36 and K38, all part of loop 3 (a.a.'s 32-41) contributed to heparin binding and cytotoxicity [35] (Figure 2). Surprisingly, when purified from granules of circulating cells, large quantities of the protein were found to lack cytotoxic activity [36]. ECP has not, like EPX/EDN, been found have alarmin activity, stimulating dendritic cells during Th2 immune responses [37], but ECP has the ability to bind lipopolysaccharide (LPS) and other bacteria cell wall components [38] which might have a priming influence on the immune system. The binding of LPS was mainly attributed to a.a.'s 1 to 45 [39]. The 1 to 45 a.a. region was found to retain bactericidal activity as well as membrane destabilization activity. One commonly occurring polymorphism in the gene is leading to the replacement of an arginine residue with a threonine, R97T [40] (Figure 2). The a.a. alteration reduced ECP cytotoxicity to the cell line NCI-H69 assessed by using both recombinant protein [36] and pools of naive protein variants [41]. RNase activity was however not influenced by the R97T alteration. Deglycosylation of the recombinant T97 restored the proteins cytotoxicity suggesting that glycosylation are responsible for this inhibitory role.

## The physiological function of the granule contained cytotoxic ribonuclease

Eosinophils contain a large amount of ECP but the question is why? What is the function of this protein? There is a constitutive baseline level of the eosinophils in many tissues and certain stimuli cause elevated production and influx of eosinophils in different organs. Moreover levels of the ECP in tissue and peripheral blood robustly correlated with the number of eosinophils present, which might be indicative that the function of ECP is also key to the role of eosinophils (see table 1). Since the discovery of ECP in 1977 [8] it has been used and evaluated as a biomarker to assess activity in various inflammatory diseases. This analysis has given indirect information of the proteins role in disease. For a comprehensive review of advantages and pitfalls of the usage of ECP as a biomarker in allergic disease see ref [42]. Furthermore, a number of *in vitro *studies have addressed the direct functional activities of the protein. Detailed following is a comprehensive review of these studies with summaries in table 1 and 2. To simplify comparison the concentrations used have been recalculated to μg/mL using the mean *M*_w _of 19.000 for the native protein (average of 16-22 kDa).

**Table 1:** ECP level in biological fluids and tissues

Biological Fluid	ECP concentration (ng/mL)	**Eosinophils (×10 **^**6**^**)/mL**
**Plasma**		
Normal value	3.5	0.104 (±0.033) [112]

Ongoing asthma/allergy	3.5	N/A [43]

*S. mansoni *infection	27	0.4 (0.2-0.8) [156]

Reactive eosinophilia with ^a ^inflammation	75	1.9 (±3.2) [112]

HES	243	19.9 (±10.9) [112]

		

**Serum**		

Normal value	7	N/A

Ongoing allergy/asthma	15	N/A [43]

*S. mansoni *infection	~62	0.163

Atopic Dermatitis inflammation	~50	0.315

Bacterial infection	~19	N/A [72]

HES	45- 198	22-58 percent of total cells [111]

Renal tumour	~30	N/A [123]

		

**BALF**		

Normal value	~4	0.2 (±0.1)

Atopic asthma (challenged)	~40	55.0 (±34.3) [97]

Drug-induced ARDS	13.8	4 percent of total cells [78]

		

**Sputum**		

Normal value	95	0.2 percent of total cells

Asthma	735	13.4 percent of total cells

Eosinophil bronchitis	604	12.4 percent of total cells [157]

Experimental Viral Day -5	119.1 (8.9-1,146)	9.3 (0-30.3) percent of total cells

Rhinovirus infection Day 2	190.6 (17.2-800) ^b)^	7.5 (0.1-34.4) percent of total cells

Day 9	157.9(27.8-800)	5.5 (0.4-23.3) percent of total cells [136]

		

**Nasal lavage**		

Normal value	3-31	N/A [158]

Allergic rhinitis	9 ± 2.4	19 (±2.1) percent of total cells

Allergic rhinitis 6 hr after allergen challenge	36.6 ± 12	56.7 (±5.8) percent of total cells [159]

		

**Nasal secretions**		

Normal value	56.2 (33.5-94.2)	

RSV infection	379 (269-532) [75]	

Natural cold	13038	

Severe community acquired bacterial sinusitis	117 704 [77]	

		

**Tears**		

Normal value	<20	1 (±0.2) cells/mm^2 ^in subepithelium [160]

Atopic keratoconjunctivitis	215 (36-1900) [161]	N/A

Vernal keratoconjunctivitis	470 (19-6000) [161]	112 (±37) cells/mm^2 ^in subepithelium [160]

		

**Skin, cutaneous**		

Normal	N/A	

Atopic dermatitis	>16 000 [64]	

ECP measurements in various biological fluids. Type of fluid, concentration of ECP measured and number of eosinophils are presented.

a) Patients with asthma, atopic dermatitis, lung disease, GI diseases, idiopathic/autoimmune inflammatory conditions

b) Statistically significant increase

**Table 2:** *In vitro *experiments analysing the activity of ECP

Cell type or other	ECP added (μg/mL)	Incubation time	Outcome compared to control	Inhibitory factors used	Reference
**Interactions with immune cells, epithelium and fibroblasts**					

					

human mononuclear cells (lymphocytes) stim. by PHA	0.2-2	48 hr	67 - 50 percent inhibition of growth		[86]

Plasma cell line	0.5 ng/mL		inhibition of Ig production	anti ECP ab	[87]

B lymphocyte cell line	1 ng/mL		inhibition of Ig production		[88]

					

Rat Peritoneal Mast Cells	17	45 min	50 percent increased histamine release		[92]

Human heart Mast cells	4.7	60 sec	10-80 percent increased histamine release PGD_2 _synthesis	Ca ^2+^, temperature	[94]

					

Guinea-pig tracheal epithelium	103	6 hr	exfoliation of mucosal cells		[79]

Feline tracheal epithelium	2.5	1 hr	release of respiratory conjugates		[99]

Human trachea	2.5				[99]

Human primary epithelial cells	10	6 hr	rECP, necrosis		[80]

Bovine mucus	100		3 fold altered structure		[97]

					

Nasal epithelial cells	2.1 ng/mL		upregulation of ICAM-1		[100]

Human corneal epithelial cells	100		decreased cell viability		[98]

Epithelial cell line NCI-H292	20 ng/mL	16 hr	upregulation of IGF-1		[102]

Human fetal lung fibroblast (HFL1)	10	48 hr	release of TGF beta, collagen contraction		[81]

Human fetal lung fibroblast (HFL1)	10	5 hr	rECP and naive, migration	anti ECPab	[107]

Human fetal lung fibroblast (HFL1)	10	6 hr	6 fold increased proteoglycan accumulation		[108]

					

**Potential effects due to high ECP levels in circulation and skin**					
					

Injection in skin intradermally	48 - 190	7 days	ulceration, inflammatory cell influx	poly lysine, MPO, onconase, carboxymethylation of RNase site, RI	[114]

Plasma	18		Influencing coagulation factor XII, shortened coagulation time		[117]

Myosin heavy chain (MHC)	16.25	8 hr	20 percent degradation of 50 ug MHC		[118]

Guinea-pig intracerebrally	0.1-30	0 - 16 days	low dose affecting cerebral activity, high dose, death		[121]

					

**Human cell lines**					

					

K562	21	4 days	50 percent inhibition of growth		[34]

HL-60	21	4 days	"		[34]

A431	76	4 days	"		[34]

					

KS Y-1	1	16 hr	29 percent decreased viability		[126]

					

HL-60	80		rECP, 50 percent inhibition of growth		[31]

HeLa	160				[31]

HeLa	320	72 h 1 hr 24 hr	50 percent inhibition of growth 4 fold increase in cytosolic Ca^2+ ^ 1.5 fold increase in Caspase like activity		[125]

					

**Interaction with pathogens**					

					

Larvae of *S. mansoni*	190		60 percent killed		[131]

Three day old *S. mansoni*	190		paralyzing		[131]

*Trypanosoma cruzi*	950	6 hr	40 percent killed		[132]

*Brugia malayi*	950	48 hr	90 percent killed		[132]

					

*Escherichia coli*	50	2 hr	72 percent decreased cfu		[135]

*Staphylococcus aureus*	50	2 hr	100 percent decreased cfu		[135]

" "	16	o.n.	rECP, 65 percent decreased cfu		[21]

					

RSV-B	9.5		rECP, 6 fold reduction in infection		[139]

ECP's influence on human cells, parasites, helminths, bacteria and viruses analysed *in vitro*. Presented in the table are amount protein used, duration of exposure, outcome and means to block the activity to prove specificity of the influence. anti ECPab: anti ECP antibody, rECP: recombinant ECP, RI: RNase inhibitor, o.n.: over night, cfu: colony forming units

## ECP during homeostasis and measured in inflammatory diseases

At homeostasis the eosinophil contributes 1 - 4 percent of the circulating leukocyte pool. ECP is readily detectable in blood with plasma levels on the average 3 ug/L (serum 7 μg/L) in healthy individuals which correlates with circulating eosinophil numbers [43]. ECP in blood shows a turnover time (t_1/2_) of 45 min [44], and the plasma protein α_2_-macroglobulin (α_2_M) is found to be associated to the protein, *in vitro *at a molar ratio of 1.6 (ECP/α_2_M). This interaction is facilitated by proteolytic activity of cathepsin G or methylamine [45], and conceivably takes place to facilitate the clearance of ECP [46].

**Figure 3:**
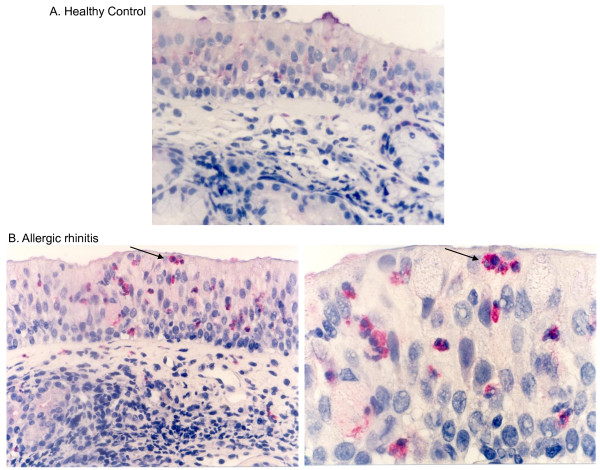
**Eosinophil granulocytes in the nasal mucosa**. (A) Immunohistochemical staining of nasal biopsy specimens for ECP in (A) a healthy control and (B, C) a patient with perennial allergic rhinitis. In healthy controls (A), a few cells are staining weakly for ECP in the submucosa and epithelium. In patients with perennial allergic rhinitis cells staining intensely for ECP are present in the submucosa and epithelium. (original magnification, ×420). (C) Higher magnification highlighting eosinophil granules in the epithelium residing cells (original magnification ×1050); Mayer's hematoxylin.

**Figure 4:**
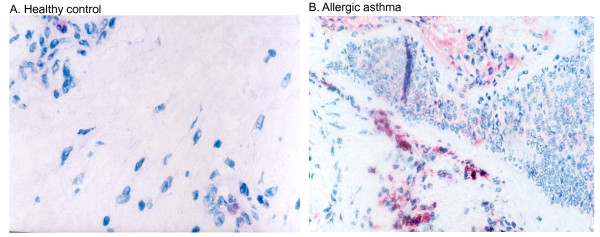
**Eosinophil granulocytes in the bronchial mucosa**. Sections of bronchial biopsies from (A) a healthy control or (B) an individual with allergic asthma were stained with ECP antibody visualizing eosinophils in the mucosa. The figures show that only a few eosinophils are present in the tissue of the healthy control, but many eosinophils accumulate in areas of reduced epithelial integrity in a specimen from a patient with allergic asthma. Original magnification ×420; Mayer's haematoxylin.

**Figure 5:**
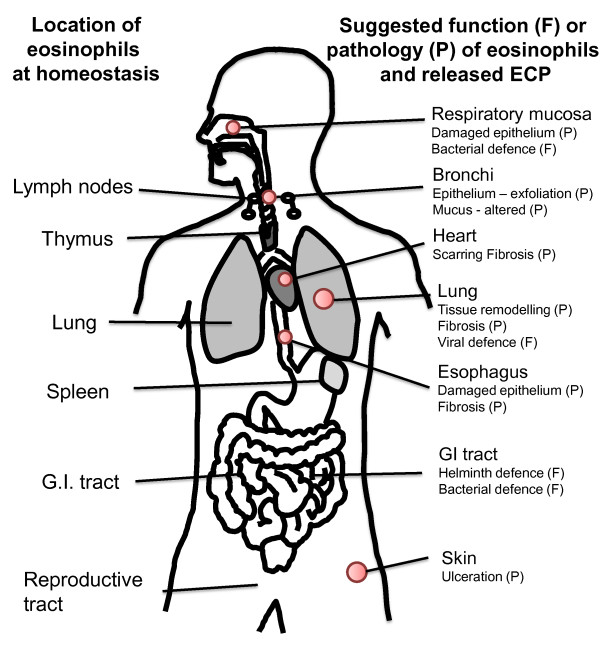
**Known anatomical locations of eosinophil granulocytes and suggested activities of released ECP at these sites**. On the left side are eosinophil granulocytes locations at homeostasis shown. On the right side are areas speculated to be affected by increased numbers of eosinophils and elevated levels of released ECP, in disease (pathology, P) and in physiological defense (function, F). This is a speculation by the authors of the review.

When eosinophils encounter adhesion molecules expressed on the endothelial cells of post capillary venule wall, the cells adhere and emigrate through the cell layer [47]. Local signals do however drive a low level influx of eosinophils in specific tissues at homeostasis. Eosinophils are present in almost all mucosal associated tissues, nasal mucosa [48] (Figure 3B), lungs [49] (Figure 4B), gastrointestinal mucosa [50], the reproductive tract, the uterus [51], breast mucosa of mice [52] and skin [53]. The chemokine eotaxin is responsible for homeostatic eosinophil influx in the gastrointestinal tract in mice [54] whereas the mechanism of influx in other organs remains unknown. In addition, lymphocyte-associated tissue: lymph nodes [50], thymus [55] and spleen [50] will have some cells stained red by eosin (see Figure 5).

The majority of ECP is released after the cell has left the circulation [56]. Several types of inflammatory stimulation have been shown to cause eosinophil degranulation. Interaction with adhesion molecules [57,58], stimulation by leukotriene B_4 _(LTB_4_), platelet activating factor (PAF) [59], interleukin (IL)-5 [60] immunoglobulins and complement factors C5a and C3a [61] all cause ECP release. Upon stimulation of eosinophils small variants of ECP with sizes 16.1 and 16.3 kDa are released [62]. One line of studies have suggested that during inflammation whole eosinophil granules are released from disrupted cells (Figure 4B) and that internal proteins are subsequently released differentially through the process of piece meal degranulation [4].

**Figure 6:**
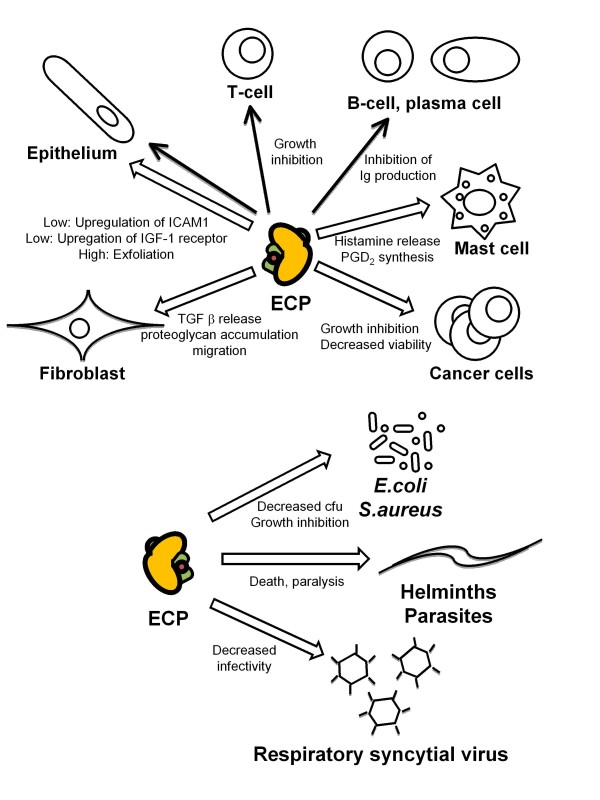
**ECP's specific influences on various cell types and micro organisms *in vitro***. Alpha helixes in the protein are shown with green color and the location of the active site is marked with a red dot. Open arrow indicates moderate (1-5 μg/mL) to high (>5 μg/mL) concentrations of ECP used in the *in vitro *experiments. Filled arrow indicates low amounts of ECP used in the *in vitro *experiments (<1 μg/mL).

Several diseases are associated with eosinophils and ECP. Most common are diseases associated with atopy and the T helper lymphocyte type 2 (TH2) phenotype. Cytokines such as IL-5 [63], or chemokines such as eotaxin are produced in elevated levels and attract elevated numbers of eosinophils to the lumen and bronchi of the lungs in asthma [49] (Figure 4B), the nasal mucosa in allergic rhinitis [48] (Figure 3B) and to the skin in atopic dermatitis [64]. In addition, the gastrointestinal tract and esophagus are infiltrated during conditions such as ulcerative colitis [65] and eosinophil esophagitis [66]. ECP has been measured in disease and the increase in number of activated eosinophils is associated with elevation of serum ECP (sECP) and plasma ECP levels [67]. Anticoagulants such as EDTA attenuate ECP release from eosinophils giving a snapshot of the *in situ *ECP level in plasma. sECP level on the other hand is often higher than plasma ECP as it's an artificial measure obtained by detection of the protein released during the blood clotting process in the test tube. sECP is thought to reflect the activation state of eosinophils [68]. ECP has also been detected in several other biological fluids such as bronchoalveolar lavage fluid (BALF), sputum, nasal lavage and in mucosa of the intestine [69]. ECP levels in various biological fluids in various diseases are presented in table 1. ECP measurements in allergic asthma have been found useful in monitoring the disease as sputum ECP correlates with forced expiratory flow (FEV) [70] and the need for glucocorticosteroid (GC) therapy while sECP correlate with eosinophil numbers in blood [71]. sECP is also elevated in some but not all cases of TH2 cytokine associated atopic dermatitis [72] eosinophil esophagitis [73], parasite infection [74] and childhood respiratory syncytial virus (RSV) infection [75]. Raised levels of ECP have also been found in some cases that are not TH2 associated; a group of patients with bacterial infections had elevated sECP [76], very high levels were found in nasal secretions from patients with bacterial sinusitis [77] and in sputum of a patient with tuberculosis and drug-induced acute respiratory distress syndrome (ARDS) [78]. Malignancies with primary eosinophilia are associated with the highest measurable sECP levels (see HES and malignancy section). Polymorphisms have been shown both to alter expression level and the function of the protein which might complicate the usage of the protein as a biomarker (see polymorphism section). The pathology attributed to eosinophils and ECP has been of both acute character such as defoliation of airway epithelium or activation of other cells [79-81] and of a chronic type, such as fibrosis in lungs [49] (Figure 5). Below we discuss the studies that indicate how ECP release influence other cell types locally (Figure 6).

### ECP and lymphocytes

Lymphocyte activation mutually with ECP level has been shown to correlate with acute exacerbations in asthma [82]. sECP is also reduced during immune therapy which is a regimen that suppresses lymphocyte activity [83]. Eosinophils have been shown to migrate to lymph nodes where they might interact with T- lymphocytes. Eosinophils up-regulate major histocompatibility complex class II [84] for antigen presentation, thereby possibly contributing to T-lymphocyte activation and the increased inflammatory response during allergic inflammation [85]. Eosinophils are also present in the lymphocyte rich organs, the thymus and spleen and lamina propria of the gastrointestinal (GI) tract [50]. Although no studies have shown any direct link between ECP release and lymphocyte function, ECP released during the inflammatory processes, co-localises with lymphocytes. *In vitro *ECP has been shown to influence the proliferation of T and B lymphocytes which indicate that the protein could regulate those cells *in vivo *(Figure 6). This was shown when mononuclear cells (containing lymphocytes, 2 × 10^5^) were incubated with or without phytohaemagglutinin (PHA) and low levels of ECP (1 nM - 0.1 μM, 190 ng/mL-2 μg/mL) for 48 hr, resulting in 50-67 percent inhibition of proliferation of the lymphocyte fraction [86]. The cells were not killed by these low levels of ECP. B lymphocyte activity might also be influenced by ECP since low levels (0.5-1 ng/mL) inhibit immunoglobulin production by plasma cells [87] and by B lymphocyte cell lines [88]. This effect was inhibited by anti-ECP antibodies and ECP was not toxic to the cell lines as cell proliferation was not inhibited with these low concentrations. IL-6 could restore the immunoglobulin production by the plasma cells and IL-4 had the same influence on the B lymphocytes. Primary human plasma cells and large activated B lymphocytes responded to ECP in a manner similar to that of the cell lines [87]. Thus, ECP might influence the immune system in that immature lymphocytes are inhibited in their proliferation by ECP while activated B lymphocytes respond by decreased immunoglobulin production (see Figure 6).

### ECP and Mast cells

Mast cells are found in the skin and in all mucosal tissues at homeostasis, and numbers are elevated in asthmatics lungs [49]. Mast cell and eosinophil numbers in mucosa are correlated to bronchial hyperactivity (BHR) [89] and mast cell products and eosinophil MBP but not ECP induces BHR [90]. Several lines of evidence suggest that there is a cross talk between eosinophils and mast cells [91] which to some extent are related to ECP release. Mast cells produce and secrete IL-5, PAF and LTB_4 _known to augment ECP release from eosinophils. Rat peritoneal mast cells on the other hand incubated with moderate levels of ECP (0. 9 μM/17 μg/mL) for 45 min released 50 percent of their histamine. Histamine is not released from peripheral basophils by ECP treatment (as by MBP) [92]. However, the release of histamine may be location specific as no release was observed from human skin mast cells treated with up to 200 μg/mL ECP [93]. Histamine and of some tryptase was though released from human heart mast cells, purified from traffic victims or from individuals undergoing heart transplantation, when stimulated with moderate levels of ECP (2.5 μM; 4.7 μg/mL). Between 10 and 80 percent of preformed mediators were released from these cells and MBP had a similar effect whereas EPX/EDN did not induce any release [94]. This ECP induced histamine release occurred within 60 sec of stimulation and was found to be Ca^2+^-, temperature- and energy dependent, and ECP was not toxic to the cells. Another mast cell product, prostaglandin D_2 _(PGD_2_) was synthesised *de novo *by the same amount of ECP added. PGD_2 _is a chemoattractant for eosinophils and TH2 lymphocytes, through binding the CRTH2 receptor [95]. Therefore these findings suggest that in some tissue the interactions between mast cells and eosinophils can be attributed to the positive feedback of ECP release.

### ECP and epithelium

ECP is detected in nasal mucosa in association with damaged epithelium [48], in damaged corneal epithelium [96] as well as in BALF (at 40 ng/mL, table 1) [97]. The function of ECP has been assessed using several assays in the view of the presence of the eosinophil in the airways. Both destructive and non-destructive consequences have been found when analyzing various concentrations of the protein in interaction with the epithelium. High levels of ECP (5.4 μM/103 μg/mL) caused exfoliation of guinea-pig mucosal cells after 6 hr incubation with tracheal epithelium [79]. Confluent primary human corneal epithelial cells incubated with 0-100 μg/mL ECP, displayed a concentration-dependent gradual increase in morphological change and with the highest concentration, 100 μg/mL, being cytotoxic [98]. Lower concentration of the ECP (2.5 μg/mL) caused release of respiratory glycoconjugates (marker of mucus secretion), with a peak after 1 hr, from feline tracheal explants [99]. The short incubation time and possibility to repeat the stimulation suggested a non-toxic mechanism. MBP, which is almost as basic as ECP, in the same assay, showed the opposite effect; therefore these effects on mucus secretion are unlikely to be due to electrostatic charge. ECP at these moderate levels (2.5 μg/mL) displayed the same effect on human trachea [99]. However human primary epithelial cells underwent necrosis at higher levels (10 μg/mL) in another study [80]. ECP has also been shown to acting directly on airway mucus *in vitro*. At high levels (100 μg/mL) ECP altered bovine mucus three fold, as measured by a capillary surfactometer [97]. At low levels ECP (0.1 nM; 2.1 ng/mL) was instead found to increase the expression of intracellular adhesion molecule (ICAM)-1 on nasal epithelial cells [100]. ECP has previously been shown to be released from eosinophils when the cells adhere with their β2 (CD18) integrins to ICAM-1. Therefore the ECP triggered up-regulation of ICAM-1 on epithelial cells might mediate a positive feedback mechanism [101]. ECP has also been proposed as a mediator of tissue remodelling, see the fibroblast section below. When low levels of the protein (20 ng/mL) were used to stimulate the bronchial epithelial cell line NCI-H292 for 16 hr, the insulin growth factor (IGF)-1 receptor was found to be up-regulated [102]. ECP was speculated therefore to be involved in IGF-1-dependent lung tissue repair processes perhaps present during homeostasis and abnormally amplified during inflammatory conditions.

### ECP and Fibroblasts

The persistent high number of eosinophils and ECP in the lungs of allergic asthmatics has led to the suggestion of their participation in the development of chronic lung tissue remodelling. Remodelling has also been found in the esophagus of patients with eosinophil esophagitis [103] and sECP has been found elevated in one case [104]. The remodelling in asthmatic lungs is in part caused by collagen and proteoglycan secretion from interstitial fibroblasts. Eosinophils have been suggested to participate in this by secretion of transforming growth factor (TGF) beta [105,106] but here is additionally described how ECP could influences fibrosis development. Stimulation of a human fetal lung fibroblast cell line (HFL1) with moderate/high levels of ECP (5-10 μg/mL) for 24-48 hr resulted in increased release of TGF-beta [81]. ECP also augmented fibroblast mediated contraction of collagen gel and stimulated migration of HFL1 fibroblasts which could be blocked with antibodies to ECP [107]. In addition, ECP incubated with the fibroblast cell line for 6 hr resulted in a 6-fold increase of intracellular proteoglycan accumulation [108].

### ECP and bronchial smooth muscle cells

Bronchial smooth muscles cells are involved during the progression of asthma development by secretion of cytokines as well as remodelling due to proliferation. Eosinophils have been found located in close proximity with smooth muscle cells. ECP does not influence smooth muscle cells by causing BHR [90] but high levels of ECP, similar to used for epithelial cells, appears to be cytotoxic, inducing cell death by *necrosis *in 1 hr. TNF alpha in contrast causes *apoptosis *of the smooth muscle cells [109].

### ECP in Hypereosinophilic Syndrome (HES)

Conditions where eosinophils are overproduced lead to detrimental effects for the host. One such condition, HES is defined by the presence of more than 1.5 × 10^6 ^eosinophils/mL blood during a time period of at least 6 months, organ involvement and with no other etiology identified. One form of HES, the myeloproliferative form, is caused by an 800 bp deletion on chromosome 4 during the haematopoiesis in the bone marrow, resulting in a fusion between the gene *FIP1L1 *and the *PDGFRA *gene [110]. A fusion protein is produced which constitutively phosphorylates tyrosine residues leading to malignant expansion of eosinophils. Another form of HES is a clonal lymphocytic variant (L-HES) where aberrant cytokine production by malignant lymphocytes causes HES. For other cases the cause of the overproduction of the eosinophils is unknown but HES is associated with high levels of ECP in plasma and serum, of up to 0.2 μg/mL [111,112]. It is not know however whether theses high levels of the protein are pathological. A few *in vitro *studies might relate to the etiologies of HES. Eosinophil infiltration of the skin of HES patients is the most common clinical manifestation [113]. Some of these patients present with erosive and ulcerative lesions and ECP was found both deposited and taken up by cells in those lesions [114]. ECP's ability to cause ulcerations in the skin has been analysed by injecting the protein intradermally into guinea pig skin, where it was found that the protein can persist there for two weeks [64] which is possibly attributed to its high stability [28]. Injections of high levels of ECP (48 and 190 μg/mL/2.5 and 10 μM) caused ulcerations which were most severe after seven days [114]. Inflammatory cells were found infiltrating the inflamed area and ECP was found taken up by cells within 48 hr. Injection of poly-lysine, other basic granule proteins MBP, EPO and the basic ribonuclease onconase showed that the severity of the lesions was not directly correlated with level of basicity. ECP and EDN were found to be more potent in lesion formations than MBP and EPO. Addition of RNase inhibitor or obliteration of the RNase activity by carboxymethylation of the RNase site of ECP reduced the ulcerations by 60 percent suggesting RNase activity is important, but not wholly responsible for the activity [114]. Some studies have shown that patients with HES have an slightly elevated risk for thrombosis formation systemically [115] and in the cardiac ventricle [116]. ECP has been shown to shortened the coagulation time for plasma which was dependent on an interaction with coagulation factor XII [117]. Eosinophils also infiltrate the endomyocardium of some patients and this has been suggested to be the cause of development of scaring in the ventricle [116]. High levels of ECP (16.25 μg/mL) degrade the muscle protein component, the myosin heavy chain *in vitro *[118] but it is not known whether ECP directly interacts with muscle fibres of the heart. The final stage is endomyocardial fibrosis in which eosinophils and ECP have been postulated to participate [119] by their influence on fibroblast function. Although a rare finding, a few patients with the myeloid form of HES have been reported to have central nervous system (CNS) manifestation [113,120]. It is not known whether ECP can reach the brain but ECPs effect on the CNS has been assayed by direct intracerebral injection. Guinea-pigs injected with ECP, showed with doses of 0.1 μg and up, cerebral symptoms up until the end of the experiment at day 16 [121]. Purkinje cells in the brain were decimated in this model, suggesting that the circulating ECP could affect the CNS of some HES patients if the protein reached the brain.

### ECP in malignancies

Eosinophils have occasionally been found to infiltrate developing tumours and have been suggested to have a role in fighting these malignancies [122]. The involvement of the eosinophils have been suggested by the finding of elevated sECP levels in patients with renal tumours (table 1) [123]. ECP assayed in urine from patients with urinary bladder tumours showed a twofold increase compared to normal's [124]. The elevated levels suggest presence of activated eosinophils in some patients with these malignancies. In the analysis of the possible involvement of ECP in tumour defence, ECP has been evaluated in respect of altering proliferation of various cell lines. The cell lines K562 and HL-60 were incubated with 1.1 uM (21 ug/mL) ECP and the cell line A431 with 4 μM (76 ug/mL) and this resulted in 50 percent inhibition of proliferation after four days. To analyse whether growth inhibition was related to positive charge or RNase activity, poly-lysine or RNase A was used with no effect [34]. ECP exists in two forms dependent on a polymorphism, R97 and T97. It was found that the T97 form had reduced capability to kill K562 and NCI-H69 cells [36]. These recombinant (r) ECPs were produced in a baculovirus system and deglycosylation restored the cytotoxic activity.

Furthermore, high levels of bacteria expressed rECP had 50 percent cytostatic effect on HL-60 and HeLa cells [31], compared to non-affected controls. ECP was found binding the surface of HeLa cells and caused cell death after 24 hr, accompanied by increases in intracellular radical oxygen species (ROS) generation and caspase 3-like activity [125]. A mix of ECP and EDN purified from urine and incubated with the Kaposi's sarcoma cell line KS Y-1 for 16 hr caused complete cell death at 0.625 μg/mL while 1 μg recombinant ECP produced in yeast and incubated with the same time span decreased the viability of the KS cell line by 29 percent. Proteins expressed in yeast lack glycosylation and the possible implications of this were speculated [126].

## ECP as a defence protein

Levels of serum ECP are elevated in TH2 engaging parasitic and helminth infections and eosinophils have long been thought to be a major defence against these types of infection. Elevated ECP have also been reported in some cases of bacterial and viral respiratory infections. Given that ECP is a cytotoxic ribonuclease, the ability of the protein to exterminate parasites, bacteria and virus *in vitro *has been extensively investigated (see also Figure 6).

### Parasite and helminth infections

Parasitic and helminthic infections drive the immune system towards TH2 cytokine production and concurrent eosinophilia. Since eosinophil infiltration in infected organs and skin is a common finding, eosinophils are thought to have a specific role in parasite killing [127]. Although, a challenged theory; the deposition of the cytotoxic protein ECP could be a mechanism by which the immune system kills off the intruders. Indeed, the eosinophilia in parasitic diseases is associated with elevated ECP in circulation (table 1) [72,128]. ECP is also found released from eosinophils in proximity to parasites in skin and lymph nodes [129,130]. The ability for ECP to kill or paralyse parasites and helminths have been analysed *in vitro *and high quantities were needed to influence the organisms. Three-hr-old larvae of *Schistosoma mansoni *were incubated with 10 μM (190 μg/mL) ECP and 60 percent were killed. *S. mansoni*, 3 days of age, were paralysed by the protein [131] while 50 μM (950 μg/mL) ECP killed 40 percent of *Trypanosoma cruzi *by 6 hr and 90 percent of *Brugia malayi *by 48 hr. This cytotoxicity of ECP to parasites was inhibited by heparin [132] and dextran sulphate, probably by interfering with the tryptophan and arginine residues as discussed earlier. In addition, heat obliterated the toxic effect of ECP to parasites, highlighting the importance of the conformation of the protein [133]. The RNase activity of ECP was clearly shown not to be important for parasite toxicity, similar to that observed for EPX/EDN.

### ECP in bacterial inflammation

Eosinophils are found lining and degranulating in both the respiratory and gastrointestinal mucosa [50]. Eosinophils are generally not thought of as defendants during bacterial inflammation. However sECP has been found elevated in septic patients [76] and very high levels of ECP in nasal secretions from patients with normal cold (13 μg/mL) or severe community acquired rhinosinusitis has been described in one case (11.7 μg/mL, table 1) [77]. Moreover, a recent study has shown that eosinophils expel mitochondrial DNA coated with ECP and other granule proteins which are bactericidal in mice *in vivo *[134]. Additionally, a few studies have described neutrophils producing ECP [15]. In view of these findings the anti - bacterial properties of ECP has been evaluated. Bacterial strains chosen for analysis were *Escherichia coli *(*E. coli*) and *Staphylococcus aureus *(*S. aureus*). High levels of ECP (50 μg/mL) decreased the number of colony-forming units (cfu) by 72 percent and close to 100 percent, respectively, for the two strains after a very short 2 hr of incubation. ECP only killed *E. coli *growing in logarithmic phase and acted on both the inner and outer membranes of *E. coli *[135]. Recombinant ECP was also cytotoxicity to *S. aureus. *Overnight incubation of rECP with the bacteria (16 kDa, 16 ug/mL/1 uM) left 35 percent of the cfu. rECP in which a.a.'s involved in RNase activity had been substituted (K38R and H128D), terminating the RNase activity, had no effect on the bacterial killing activity [21]. In conclusion therefore, eosinophils and ECP might have a role in bacterial defence. Due to its stability, it might be feasible to speculate that ECP over time accumulate in mucus fluids such as nasal secretions and act as a first line of defence against bacterial intrusion.

### ECP in viral inflammation

ECP has been found significantly elevated in sputum from atopic subjects subjected to experimental rhinovirus infection [136] and in nasal secretions from atopic infants with respiratory RSV infection (table 1) [75]. Eosinophils and ECP are associated with RSV infection in children's lungs [137] and RSV can infect, and replicate in eosinophils [138]. Recombinant ECP expressed in a baculovirus system was used to evaluate whether ECP can inactivate the B subtype of RSV. ECP (0.5 μM; 9.5 μg/mL) incubated with the virus showed a 6-fold reduction of the infectivity of the virus to a human pulmonary epithelial cell line [139]. This antiviral activity was lower than that found with EPX/EDN (54-fold reduction) [140], but the infectivity was increased by addition of RNase inhibitor (RI) to both proteins during incubation. Mixing the two proteins did not mediate any synergistic effects on antiviral activity. RNase A, however [up to 4 mM (76 mg/mL)], did not exert antiviral activity, suggesting that the RNase site but not activity is important for inhibition of infectivity.

## Polymorphisms in the *RNASE3 *gene and association to production and disease

Table 3 summarizes data from the NCBI entrez nucleotide site regarding polymorphisms detected in the ECP gene. Two polymorphisms are found in the protein coding region, two in intronic regions and two in the 3' untranslated region (UTR). ECP polymorphisms are differentially distributed according to ethnicity [141]. Two studies have evaluated polymorphisms in intronic and UTR regions of the ECP gene, and linked them with ECP production. One polymorphism rs11575981 (-393T > C) located in the promoter, in an C/EBP binding site was associated with decreased ECP level in serum, and decreased binding of C/EBP alpha [142]. Another polymorphism, in the 3'UTR, rs2233860 (499G > C or 562G > C) was associated with content of ECP in the eosinophils [143]. Three studies have analysed whether any polymorphisms are linked to allergic asthma and allergic rhinitis. The presence of the C allele in the nonsynonymous rs2073342G/C (371G > C/434G > C) polymorphism in the ECP gene, causing a.a. alteration Y97T, was found to be associated with absence of asthma in one Swedish study [40]. A study of Norwegian and Dutch subjects instead found that the haplotype C-G-G for the three polymorphisms rs2233859/rs17792481 (-38C/A), rs2073342 (371G/C/434G/C) and rs2233860 (499G/C/562G/C) being protective [144]. In a third, Korean study, which was the largest, the genotype rs2233860CC (499/562CC) was associated with allergic rhinitis [145]. Eosinophils occasionally infiltrate oral squamous cell carcinoma tumours. A study found a tendency for association of the rs2073342G/C C/C (371/434GC/CC) genotypes with a poor clinical outcome in patients with eosinophil rich such tumours [146]. As discussed earlier, eosinophils are present during helminth infections. The rs8019343 polymorphism T (1088TT) in the 3'UTR was exclusively present in the genome of a patient with tropical pulmonary eosinophilia [147]. Furthermore a study has found the rs2073342 with C (371/434C) polymorphism overrepresented in helminth infected Ugandans [148]. Interestingly, from the -550 polymorphism over a stretch of 272 bases to the mRNA transcription start site, thirteen polymorphism sites are located (NCBI Reference Sequence: NC_000014.7, J. Bystrom unpublished observation). Similar to the protein coding region and the 3'UTR, this region is highly homologous to the *RNASE2 *gene region, with the only differences being the sites of the polymorphisms. The replacement base's for twelve of the thirteen polymorphisms is to the same base as in the *RNASE2 *promoter sequence. This is also the case for two of the 3'UTR polymorphisms. This further highlights the extremely close relationship between *RNASE3 *(ECP) and *RNASE2 *(EPX/EDN).

**Table 3:** Polymorphisms associated with the *RNASE3 *gene

Polymorphism	alleles	Alternative names	location, effect
**rs2284954**	A/G	-550A > G	promoter

**rs11575981**	C/T	-393T > C	promoter, disrupt C/EBP binding site, correlate with s-ECP [142]

**rs2233858**	C/T		intron

**rs2233859/rs17792481**	A/C	-38C > A	intron (in a GATA-1 site) ^a^

**rs2073342**	C/G	371G > C, 434G > C	protein coding, Y > G is associated with allergic asthma [40]^a^, poor outcome in oral squamous cell carcinoma tumours [146], C over represented in helminth infected Ugandans [148]

**rs12147890**	A/G		protein coding

**rs2233860**	G/C		3' UTR,G is correlated to higher intracellular ECP [143], G is associated with allergic rhinitis [145], ^a^

**rs8019343**	A/T	499G > C, 562G > C	3' UTR T is only present in one patient with helminth infection [147]

Polymorphisms found in the ECP gene and surrounding chromosomal sequence. Listed are Polymorphism i.d.'s, altered bases, alternative names, and types of associations

a) C-G-G haplotype associated with allergic rhinitis [144]

## Discussion

ECP was first discovered in 1977 and since then, evidence has been gathered to understand its roles in physiology and pathophysiology. ECP is a peptide of 133 a.a., with the first 40 a.a. necessary for membrane interfering, heparin binding and cytotoxic activity. The heparin binding ability of ECP might enable the protein to bind proteoglycans on other human cells for possible uptake [34] or heparan sulfate in extracellular matrix for later use such as is the case for CXCL10 [149]. In a similar manner ECP might bind microorganisms peptidoglycans for uptake and cytotoxicity [32]. The non-synonymous polymorphism rs2073342 reduces cytotoxicity suggesting an alteration of the three-dimensional structure influencing catalytic site elsewhere in the protein. ECP is glycosylated, and as recently discovered can be nitrated. The development of increasingly sophisticated assays will determine whether other modifications, perhaps function associated, are also important in ECP activity.

Since the discovery of ECP, assays have been developed to determine its levels in biological fluids in various diseases (table 1). ECP in serum can reach 0.1 - 0.2 μg/mL for HES patients [111] and parasitic diseases infected individuals [72] and this is a 30 fold elevation compared to ECP in serum of healthy individuals. In BALF and nasal lavage from atopic patients the ECP levels are lower, 0.050 μg/mL but the sample are diluted during the collection process. In undiluted tears, sputum and nasal secretions the highest ECP levels have been found: 0.5, 0.7 and 10 μg/mL, respectively. The ECP measurements correlate with eosinophilic disease but have been found elevated also in some diseases without known eosinophil involvement [76-78]. The biological activity of ECP has been studied by incubation of the protein with several different cell types *in vitro*. Both human cells and pathogens have been assayed analysing different parameters (see table 2). In general, 10 - 20 μg/mL and above, result in growth inhibitory and destructive consequences to mammalian cells, parasites and bacteria. ECP released *in situ *in diseases engaging high levels of eosinophils might reach these destructive concentrations (e.g. ECP accumulated in air way mucus of asthmatics, in nasal secretions of some sinusitis patients or released in skin of atopic dermatitis/HES patients, table 1 and Figure 5). Although it remains to be proven, there is a possibility that destructive activity to multiple cell types as well as induction of fibrosis is part of the etiology of disease where ECP levels are elevated during prolonged periods, e.g. in HES and helminth infection. There is also evidence that neutrophils are carriers of significant amounts of ECP. Using the murine system, granule proteins have been found associated with expelled eosinophil mitochondrial DNA and this DNA/protein complex trapping and killing bacteria in the gut [134]. It is intriguing to speculate whether the high levels of ECP present in various human mucosal secretions would equally be associated with eosinophil mitochondrial DNA and whether such complexes had the ability to capture and kill microorganisms.

The role of eosinophils in asthma has been under scrutiny since clinical trials showing that anti-IL-5 therapy did not improve the disease symptoms for allergic asthmatics albeit eosinophil numbers were reduced [150]. However, two recent clinical trials have shown that anti-IL-5 antibodies actually could relieve symptoms in eosinophil rich, late onset asthma, suggesting that eosinophils can have a pathogenic role in this disease. In these trials inflammatory exacerbations were reduced when anti-IL-5 antibodies were administrated [151,152]. Earlier studies using diagnostic ECP measurements seem to agree with these findings as ECP levels correlate with severity of asthma: FEV (sputum ECP) [70], need for GC treatment (sputum ECP) [153] and blood eosinophilia (sECP) [71]. Results from *in vitro *studies presented in this review may well suggest several roles for ECP in this type of allergic asthma. The protein might act as an inflammatory amplifier by augmentation of release of, for eosinophils chemotactic, PGD_2 _from mast cells in asthmatic patients. Moreover, protein released in the interstitium might influence fibrosis development (Figure 3B, 4B and 5). One might speculate that blocking antibodies to ECP could be a symptom relieving addition to the already established GC and anti-IL5 therapies used in eosinophil rich asthma and other eosinophilic diseases [113,151,152].

Table 2 shows that the level of protein needed to influence proliferation of lymphocytes and their antibody production is 1000 times lower than the destructive levels described above, i.e. in the ng/mL range. In murine system eosinophils have been ascribed a novel role in inflammation; the cells enter and contribute to the well orchestrated process of inflammation resolution of by release of the pro-resolving lipid protectin D1 [154] (for a review see [155]). Whether ECP is released during this resolution process for the dual role of sequestering subpopulations of inflammatory lymphocytes [86] and promoting tissue repair by TGF beta augmentation [81] is an intriguing speculation. Eosinophils are also present at homeostasis at low numbers in lymphocyte rich organs at various locations but degranulated only in the GI tract [50]. A single eosinophil contains 13 pg ECP. Do eosinophils have a role in maintaining homeostasis and do low levels of ECP also have a role here? EDN, the sister protein has been found to play an active role during inflammation development influencing the maturation of DC's [37]. If EDN is pro-inflammatory, perhaps the two proteins divergence could be because ECP might have acquired a novel role as yet unknown role.

Finally, analysis of the DNA sequence of the ECP gene and surrounding regions have unravelled a number of polymorphisms. These studies have linked different polymorphisms and haplotypes to TH2 diseases, asthma, and allergic rhinitis. The studies have in some cases come to different conclusions but used different patients and different ethnic groups which might explain the variations. Diseases such as allergic asthma are multifactoral and to determine the role of certain polymorphisms one might need to look at larger defined groups to get a clear association. Altered expression levels might also influence both destructive functions and possible homeostatic roles. A careful analysis using all polymorphisms and corresponding haplotypes and large groups of defined populations would more clearly determine the role of ECPs genetic make-up, and its potential functions in physiology and disease.

## Conclusion

The eosinophil granulocyte was discovered 130 years ago but its roles are still being revealed. The most characteristic feature of the eosinophil is the large secondary granules filled with basic proteins. The purpose of these proteins is still not fully understood. One of the proteins, ECP is a highly basic, cytotoxic, heparin binding ribonuclease that seems to need its ribonuclease site but not activity for its activities. Sensitive assays have been developed for its measurement in biological fluids which have contributed to the understanding of the role of the eosinophils in disease. *In vitro *studies have shown that high levels of ECP are necessary for development its destructive actions. Diseases engaging high levels of eosinophils might reach these levels locally in the tissue. At those high levels polymorphisms altering expression level and protein sequence might play a role within certain populations. Whether ECP also has roles at lower concentrations, such as the growth inhibitory influences on lymphocytes found *in vitro*, remain to be shown with *in vivo *models or clinically. These additional roles for ECP when discovered, might provide critical answers to the functions of eosinophil granulocytes and is therefore well worth waiting for.

## Competing interests

The authors declare that they have no competing interests.

## Authors' contributions

JB, DBB and KA have together drafted and completed the manuscript. KA provided histological images; JB and DBB have provided other figures. All authors have read and approved the final version of the manuscript.
